# Circulating Tsukushi Predicts Incident Sarcopenia and Functional Decline in Older Adults: A Prospective Cohort Study

**DOI:** 10.1002/jcsm.70423

**Published:** 2026-11-08

**Authors:** Ming‐Qing He, Xue‐Ning Dai, Mian Zhang, Yue Yu, Liang Zhou, Jing Wu, Wen‐Ya Zhao, Die‐Jing Niu, Can Zhao, Jin‐Shui Xu, Zheng‐Kai Shen, Xiang Lu, Bin Xue, Wei Gao

**Affiliations:** ^1^ Department of Geriatrics The First Affiliated Hospital of Soochow University Suzhou China; ^2^ Translational Medicine Research Center and Jiangsu Key Laboratory of Pediatrics Children's Hospital of Nanjing Medical University Nanjing China; ^3^ Department of Laboratory Medicine Jurong Hospital Affiliated to Jiangsu University Zhenjiang China; ^4^ Department of Geriatrics, Sir Run Run Hospital Nanjing Medical University Nanjing China; ^5^ Laboratory of Anatomy, Biomechanics and Organogenesis, Faculty of Medicine Université Librede Bruxelles (ULB) Brussels Belgium; ^6^ Department of General Surgery The Second People's Hospital of Changzhou, the Third Affiliated Hospital of Nanjing Medical University Changzhou China; ^7^ Collaborative Innovation Center for Cancer Personalized Medicine Nanjing Medical University Nanjing China; ^8^ Jiangsu Provincial Key Laboratory of Biological Therapy for Organ Failure Nanjing Medical University Nanjing China; ^9^ Geriatrics Department, Nantong First People's Hospital Southeast University Nantong China; ^10^ Department of Geriatrics, Zhongda Hospital, School of Medicine Southeast University Nanjing China; ^11^ Jiangsu Province Center for Disease Control and Prevention Nanjing China

**Keywords:** older adults, sarcopenia, serum, TSK

## Abstract

**Background:**

Sarcopenia is a major age‐related condition associated with functional decline, disability and mortality in older adults, yet reliable circulating biomarkers for early risk stratification remain limited. Tsukushi (TSK), a secreted hepatokine involved in systemic metabolic regulation, has recently been implicated in skeletal muscle homeostasis. However, prospective clinical evidence linking circulating TSK to sarcopenia development is lacking. We therefore investigated the association between serum TSK levels and incident sarcopenia in a community‐based cohort of older adults.

**Methods:**

A total of 1370 community‐dwelling individuals aged ≥ 65 years were followed for 4 years. Baseline serum TSK levels were measured using enzyme‐linked immunosorbent assay. Multivariable Cox proportional hazards models were used to assess the association between TSK and incident sarcopenia after adjustment for confounding factors. Restricted cubic spline analyses were applied to explore dose–response relationships, and predictive performance was evaluated using time‐dependent receiver operating characteristic (ROC) curves.

**Results:**

Lower baseline TSK levels were associated with a higher cumulative incidence of sarcopenia. In multivariable analyses, higher TSK levels were associated with a lower risk of incident sarcopenia (hazard ratio per SD increase = 0.64, 95% CI = 0.53–0.76, *p* < 0.001). Dose–response analyses demonstrated a non‐linear association with overall sarcopenia risk, whereas associations with low muscle strength and low physical performance were more nearly linear. The association appeared more evident in women than in men. TSK alone showed moderate discrimination for incident sarcopenia (AUC = 0.700), while a combined clinical model improved discrimination (AUC = 0.808 at 4 years).

**Conclusions:**

Lower circulating TSK levels are associated with a higher risk of incident sarcopenia in older adults, particularly subsequent muscle‐related functional decline. TSK may be a candidate biomarker for early sarcopenia risk stratification and may reflect systemic metabolic processes relevant to muscle health.

## 1 Introduction

Sarcopenia is a geriatric syndrome characterized by a reduction in skeletal muscle mass, a decline in muscle strength and impairment of physical function [1]. Compared with healthy populations, the risks of falls, disability and death are significantly increased in patients with sarcopenia. With the rapid aging of populations worldwide, sarcopenia has become a severe public health challenge for global public health systems [1]. Although evaluation tools for sarcopenia are increasingly improved; however, due to the differences in age, sex, disease status, degree of cooperation, and so forth, the accuracy and sensitivity of current diagnostic criteria are still unsatisfactory [2]. It is therefore a great priority to identify feasible objective biomarkers for early screening of subjects at high risk of sarcopenia.

Tsukushi (TSK) is a small secreted leucine‐rich repeat proteoglycan (SLRP) that functions as an extracellular signalling modulator [3]. TSK was originally identified as a developmental regulator via interacting with multiple extracellular ligands and signalling pathways, including transforming growth factor‐β (TGF‐β), fibroblast growth factor (FGF), Wnt and bone morphogenetic protein (BMP) signalling, thereby modulating diverse biological processes such as cell differentiation, morphogenesis and tissue development [3]. In addition to its developmental roles, emerging evidence has suggested that TSK also participates in systemic metabolic regulation [4]. TSK is predominantly produced by the liver and released into the circulation in response to metabolic stressors such as obesity, inflammation and endoplasmic reticulum stress [4]. Genetic deletion of TSK in mice enhances thermogenesis and protects against diet‐induced obesity, supporting a role of TSK as a feedback signal that restrains excessive energy dissipation [5]. Interestingly, recent work has revealed a direct role for TSK in skeletal muscle biology [6]. Experimental study demonstrated that TSK is required for maintaining skeletal muscle mass, strength and endurance capacity [6]. In mice lacking TSK, reduced grip strength, impaired exercise performance and significant alterations in skeletal muscle gene expression were observed [6]. Despite these emerging insights, the role of TSK in human skeletal muscle disorders remains poorly understood. Importantly, circulating TSK is readily detectable in blood and has been proposed as a biomarker in metabolic disorders and other disease states [7, 8, 9, 10]. Given that systemic metabolic dysregulation and abnormal interorgan communication are increasingly recognized contributors to skeletal muscle dysfunction [11], these findings suggest that circulating TSK may represent a novel metabolic factor associated with skeletal muscle health and the development of sarcopenia. Therefore, the present study aims to investigate the association between serum TSK levels and the risk of sarcopenia in older adults.

## 2 Methods and Materials

### 2.1 Study Participants

A total of 1370 older adults aged ≥ 65 was recruited consecutively from Jiangsu Province, China, as previously described [12] (Figure 1). Participants with the following conditions were excluded: (a) bedridden or unable to move independently; (b) unable to perform specific movements due to neurological disorders, bone and joint diseases or cardiorespiratory insufficiency; (c) severe renal insufficiency (creatinine clearance < 60 mL/min) or severe hepatic damage (transaminase elevated more than two times); and (d) malignant tumours. This study was conducted under the principles outlined in the Declaration of Helsinki [13] and approved by the Ethics Committee of Sir Run Run Hospital, Nanjing Medical University (Approval Number 2019‐SR‐S041). Written informed consent was obtained from all enrolled participants.

**FIGURE 1:**
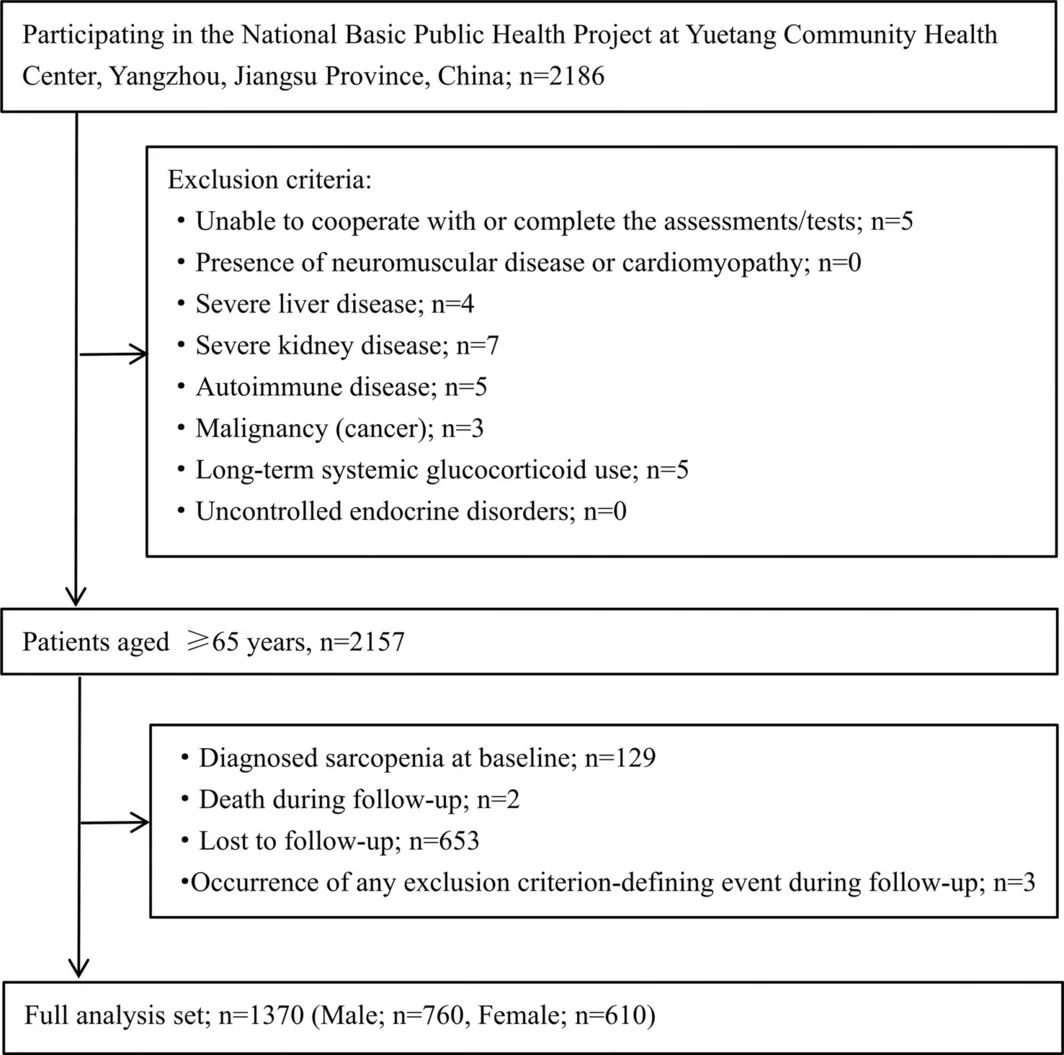
Inclusion flow of study participants.

### 2.2 Data Collection

After overnight fasting, venous blood was collected in the early morning and separated into serum and cell components within 2 h. The serum was stored at −80°C for further analysis. A series of blood biochemical indicators were measured, as mentioned previously. Participants who smoked more than one cigarette per day in the past 12 months were classified as current smokers. Current drinkers were those who drank at least once a day in the past 12 months [14].

### 2.3 Assessment of Sarcopenia

Sarcopenia was diagnosed based on the latest criteria of the Asian Working Group on Sarcopenia (AWGS) 2019 [15]. Muscle mass was measured by bioelectrical impedance analysis (BIA) using Inbody S10 (Inbody, Korea). Appendicular skeletal muscle mass index (ASMI) was calculated as ASM divided by height in square metres (ASM/height^2^). Grip strength was measured with a dynamometer (CAMRY EH101, China). Gait speed, usually on a 6‐m track, was tested for physical performance. Briefly, elderly patients with low muscle mass (ASMI < 7.0 kg/m^2^ in men and < 5.7 kg/m^2^ in women) and low muscle strength (grip strength < 28 kg in men and < 18 kg in women) and/or low physical function (walking speed < 1.0 m/s) were categorized as having sarcopenia [15]. Patients with low muscle mass combined with low muscle strength or low physical performance were assumed to have moderate sarcopenia. Patients with low muscle mass, low muscle strength and low physical performance were considered to have severe sarcopenia [15].

### 2.4 Serum TSK Measurements

Serum TSK levels were measured using an enzyme‐linked immunosorbent assay (ELISA) kit (Cat. ELH‐TSKU, RayBiotech, USA) according to the manufacturer's protocol. The intra‐assay and interassay coefficients of variance were 4.17% and 3.25%, respectively. The minimum detectable dose was 0.63 ng/mL.

### 2.5 Statistical Analysis

Statistical analyses were performed using R Version 4.5.1. Follow‐up time was expressed in years. The Kolmogorov–Smirnov test was used to test the normality of continuous variables, described as the median (interquartile range [IQR]). The Mann–Whitney *U* test was utilized to determine differences between two groups. The Pearson *χ* ^2^ test was used to compare qualitative variables expressed as frequencies. Model performance was evaluated based on the optimal cut‐off determined by maximally selected rank statistics and the Youden index, model fit assessed using the Akaike information criterion (AIC) and predictive accuracy measured by the concordance index (C‐index). Survival analysis was performed using the Kaplan–Meier method based on serum TSK levels, and differences between groups were assessed using the log‐rank test. Multivariable Cox models were used to calculate hazard ratios (HRs) and 95% confidence intervals (CIs). Sensitivity analyses were performed by repeating the primary Cox model after separately excluding participants with baseline diabetes and those with a BMI ≥ 25 kg/m^2^. Additional models adjusted for baseline ASMI, handgrip strength and gait speed, both individually and simultaneously, to assess the robustness of the associations. Subgroup analyses, restricted cubic spline analyses and time‐dependent receiver operating characteristic (ROC) curve analyses were conducted to evaluate the predictive performance of serum TSK for the study outcomes. All tests were two sided, and *p* < 0.05 was considered statistically significant.

## 3 Results

### 3.1 Baseline Characteristics

A total of 1370 participants (mean age 76 ± 7 years; 55% men) were included and categorized into tertiles of serum TSK (Table 1 and Figure 2A). Baseline age and sex distribution were comparable across TSK tertiles (*p* = 0.125 and *p* = 0.617, respectively). Residence area differed significantly, with a higher proportion of urban participants in Tertile 1 (44.3%) and Tertile 3 (41.1%) than in Tertile 2 (25.6%) (*p* < 0.001). Current smoking was more prevalent in Tertile 1 (21.5%) than in Tertile 2 (13.8%) and Tertile 3 (16.2%) (*p* = 0.007), whereas alcohol drinking did not differ significantly across groups (*p* = 0.057). The prevalence of hypertension and diabetes differed across TSK tertiles (*p* = 0.004 and *p* = 0.019, respectively). However, the prevalence of neither condition increased progressively across TSK tertiles. Both hypertension and diabetes were most prevalent in Tertile 3, whereas their prevalences were lower in Tertile 2 than in Tertile 1 (hypertension: 42.3%, 35.2% and 45.7%; diabetes: 16.2%, 14.4% and 21.2% in Tertiles 1, 2 and 3, respectively). In contrast, ASMI and gait speed increased across TSK tertiles, from 7.33 to 7.85 kg/m^2^ and from 1.02 to 1.09 m/s, respectively (both *p* < 0.001). Handgrip strength did not differ significantly across groups (*p* = 0.924). Fasting glucose, ALT, renal function indices and lipid profiles were also comparable across tertiles, whereas AST and hs‐CRP differed significantly (*p* = 0.034 and *p* < 0.001, respectively). Overall, cardiometabolic and inflammatory characteristics did not exhibit a consistent gradient across TSK tertiles. In correlation analyses, serum TSK levels were positively correlated with ASMI (*R* = 0.226, *p* < 0.001) and gait speed (*R* = 0.140, *p* < 0.001) but not with grip strength (*R* = −0.008, *p* = 0.764) (Figure 2B–D).

**TABLE 1:** Baseline characteristics of study participants stratified by tertiles of TSK levels.

Characteristic	Overall (*n* = 1370)	Low (*n* = 456)	Intermediate (*n* = 457)	High (*n* = 457)	*p*
Age, years	75.8 ± 6.5	75.5 ± 6.2	75.6 ± 6.6	76.3 ± 6.7	0.125
Female, *n* (%)	610 (44.5)	199 (43.6)	199 (43.5)	212 (46.4)	0.617
Urban area, *n* (%)	507 (37.0)	202 (44.3)	117 (25.6)	188 (41.1)	< 0.001
BMI, kg/m^2^	23.10 ± 3.17	22.87 ± 2.56	23.18 ± 3.35	23.25 ± 3.50	0.152
Current smoking, *n* (%)	235 (17.2)	98 (21.5)	63 (13.8)	74 (16.2)	0.007
Current drinking, *n* (%)	156 (11.4)	40 (8.8)	53 (11.6)	63 (13.8)	0.057
Hypertension, *n* (%)	563 (41.1)	193 (42.3)	161 (35.2)	209 (45.7)	0.004
Diabetes, *n* (%)	237 (17.3)	74 (16.2)	66 (14.4)	97 (21.2)	0.019
FBG, mmol/L	6.01 ± 1.78	5.98 ± 1.63	5.97 ± 1.72	6.08 ± 1.97	0.610
ALT, U/L	17.56 ± 10.45	17.67 ± 9.75	17.64 ± 11.84	17.36 ± 9.63	0.883
AST, U/L	24.18 ± 9.17	24.12 ± 8.92	25.00 ± 10.64	23.43 ± 7.66	0.034
TBIL, μmol/L	13.72 ± 6.07	13.48 ± 5.01	13.76 ± 5.52	13.93 ± 7.40	0.523
eGFR, mL/min/1.73 m^2^	83.92 ± 13.37	84.81 ± 13.80	83.59 ± 13.30	83.38 ± 12.97	0.217
BUN, mmol/L	5.59 ± 1.48	5.60 ± 1.41	5.52 ± 1.58	5.66 ± 1.46	0.355
TC, mmol/L	4.84 ± 0.89	4.82 ± 0.91	4.84 ± 0.93	4.86 ± 0.84	0.727
TG, mmol/L	1.25 (0.98–1.71)	1.26 (1.02–1.68)	1.20 (0.96–1.66)	1.24 (0.95–1.75)	0.637
LDL, mmol/L	2.33 ± 0.71	2.36 ± 0.77	2.32 ± 0.67	2.31 ± 0.68	0.556
HDL, mmol/L	1.45 ± 0.29	1.43 ± 0.29	1.45 ± 0.28	1.46 ± 0.30	0.357
ALB, g/L	44.60 ± 7.66	43.94 ± 7.49	45.17 ± 7.85	44.68 ± 7.60	0.052
hs‐CRP, mg/L	4.25 ± 1.82	4.33 ± 1.79	3.83 ± 1.77	4.59 ± 1.83	< 0.001
TSK, ng/mL	98.35 (73.54–116.27)	49.01 (38.02–73.41)	98.35 (93.67–105.09)	123.56 (116.31–130.87)	< 0.001
ASMI, kg/m^2^	7.63 ± 1.34	7.33 ± 1.63	7.70 ± 1.20	7.85 ± 1.08	< 0.001
Grip strength, kg	29.40 ± 7.93	29.30 ± 8.03	29.51 ± 7.97	29.39 ± 7.80	0.924
Gait speed, m/s	1.05 ± 0.16	1.02 ± 0.16	1.05 ± 0.16	1.09 ± 0.14	< 0.001

Abbreviations: ALB, albumin; ALT, alanine transaminase; ASMI, appendicular skeletal muscle mass index; AST, aspartate aminotransferase; BMI, body mass index; BUN, blood urea nitrogen; eGFR, estimated glomerular filtration rate; FBG, fasting blood glucose; HDL, high‐density lipoprotein cholesterol; hs‐CRP, high‐sensitive C‐reactive protein; LDL, low‐density lipoprotein cholesterol; TC, total cholesterol; TG, triglyceride; TSK, Tsukushi.

**FIGURE 2:**
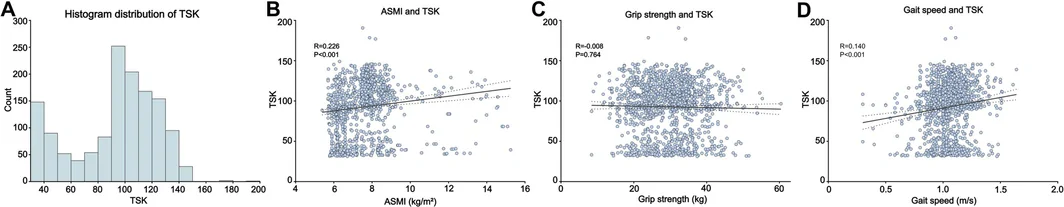
Baseline distribution of serum TSK concentrations. (A) Histogram of serum TSK levels. (B–D) Scatter plots for the associations between serum TSK levels and ASMI (B), grip strength (C) and gait speed (D). Linear regression lines and 95% confidence intervals are shown. ASMI, appendicular skeletal muscle index; TSK, Tsukushi.

### 3.2 Association of Serum TSK With the Risk of Sarcopenia

The cumulative incidence of sarcopenia differed significantly across the three TSK tertiles (log‐rank *p* < 0.001, Figure 3A), with the highest incidence observed in the lowest TSK group. Similar patterns were observed for the individual components of sarcopenia, with participants in the lowest TSK tertile having the highest cumulative incidence of low muscle mass (Figure 3B), low grip strength (Figure 3C) and low gait speed (Figure 3D) (all log‐rank *p* < 0.001). Univariable Cox regression results are presented in Table S1. In multivariable analyses, higher baseline serum TSK levels were associated with a lower risk of incident sarcopenia (adjusted HR per SD increase = 0.64, 95% CI = 0.53–0.76, *p* < 0.001; Table 2). This association remained robust in sensitivity analyses. After excluding 237 participants with baseline diabetes, 108 incident sarcopenia events occurred among the remaining 1133 participants, and the inverse association persisted (adjusted HR per SD increase = 0.63, 95% CI = 0.52–0.77, *p* < 0.001; Table S2). Further adjustment for baseline ASMI, handgrip strength and gait speed, either individually or simultaneously, yielded similar estimates. In the model simultaneously adjusted for all three baseline muscle measures, the adjusted HR per SD increase in TSK was 0.64 (95% CI = 0.53–0.77, *p* < 0.001; Table S3). Subgroup analyses showed generally consistent associations across strata defined by age, residential area and comorbidities, with no significant evidence of interaction (*p* for interaction > 0.05). A significant interaction was observed for BMI (*p* for interaction < 0.001), with a stronger inverse association between TSK levels and incident sarcopenia among participants with a BMI < 25 kg/m^2^. The estimate for participants with a BMI ≥ 25 kg/m^2^ was highly uncertain because of the small number of incident events (*n* = 10) (Figure 4). In a sensitivity analysis excluding participants with a BMI ≥ 25 kg/m^2^, 1005 participants with 116 incident sarcopenia events remained. The inverse association still persisted (adjusted HR per SD increase = 0.57, 95% CI = 0.47–0.69, *p* < 0.001; Table S4). Moreover, the inverse association also appeared more pronounced in women than in men (Figure 4); however, this subgroup finding should be interpreted cautiously.

**FIGURE 3:**
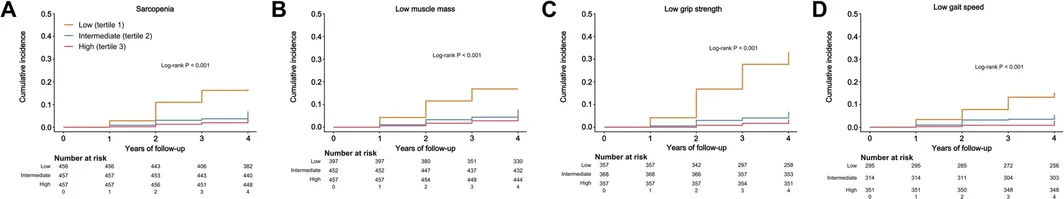
Kaplan–Meier curves for the cumulative incidence of sarcopenia (A), low muscle mass (B), low muscle strength (low grip strength) (C) and low physical performance (low gait speed) (D) in older adults according to tertiles of serum TSK levels. ASMI, appendicular skeletal muscle mass index; TSK, Tsukushi.

**TABLE 2:** Hazard ratios (HRs) for incident sarcopenia.

Variables	Univariate		Multivariate^a^	
	HR (95% CI)	*p*	HR (95% CI)	*p*
TSK, ng/mL				
Per unit	0.99 (0.99–1.00)	< 0.001	0.99 (0.98–0.99)	< 0.001
Per SD	0.74 (0.63–0.87)	< 0.001	0.64 (0.53–0.76)	< 0.001

Abbreviation: TSK, Tsukushi.

^a^ Adjusted for sex, area, BMI, hypertension, ALT, LDL, HDL, ALB and diabetes.

**FIGURE 4:**
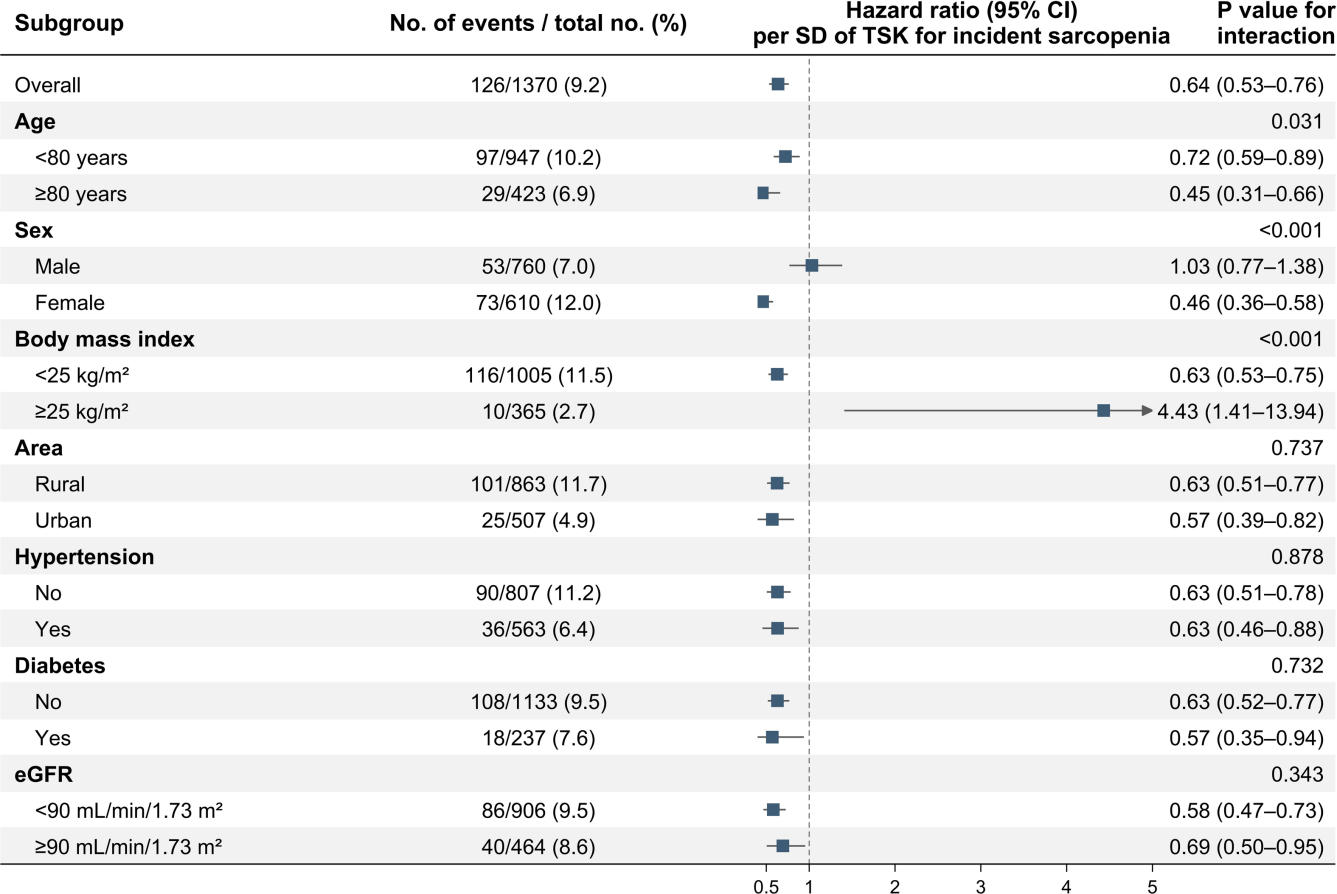
Subgroup analysis of the association between serum TSK levels and incident sarcopenia. eGFR, estimated glomerular filtration rate; TSK, Tsukushi.

### 3.3 Predictive Performance of Serum TSK

The cut‐off values were 87.21 (maximum selection rank statistics) and 100.63 ng/mL (Youden index). Time‐dependent ROC analysis demonstrated that TSK alone (Model 1) could predict the incidence of sarcopenia with an AUC of 0.700. Incorporation of clinical variables with TSK (Model 2) substantially improved predictive performance, achieving the highest accuracy at the 4‐year follow‐up (AUC = 0.808) (Figure 5A). Time‐dependent AUC curves further indicated that the predictive performance of Model 2 remained consistently high throughout the entire follow‐up period (Figure 5B).

**FIGURE 5:**
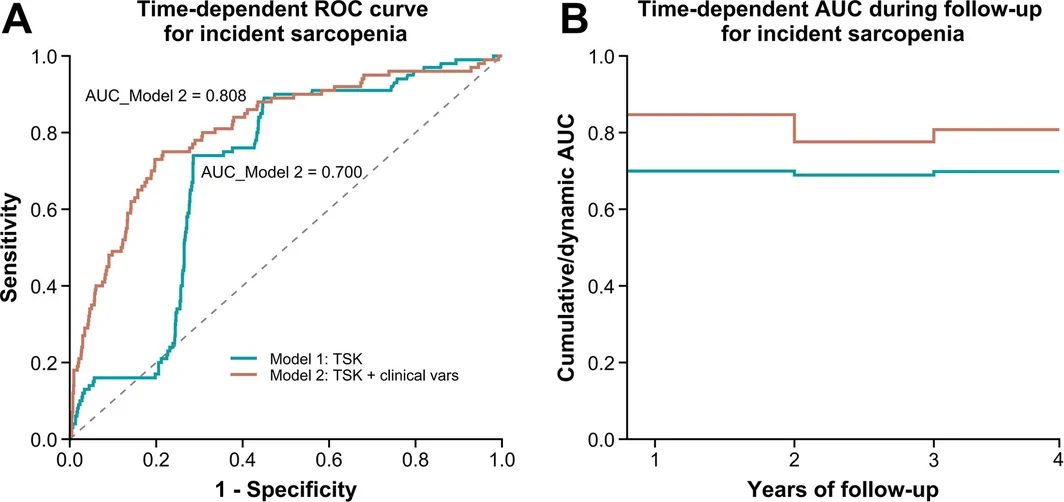
Time‐dependent ROC curve (A) and AUC (B) for the time‐dependent ROC curve during the follow‐up period for incident sarcopenia: Model 1, serum TSK; Model 2, multivariate model adjusted for serum TSK, sex, area, BMI, hypertension, ALT, LDL, HDL, ALB and diabetes.

### 3.4 TSK–Sarcopenia Association Dynamics

Restricted cubic spline analyses demonstrated a non‐linear correlation between baseline serum TSK levels and incident sarcopenia (Figure 6A) (*p* for non‐linearity < 0.001). Component‐specific analyses further showed heterogeneous association patterns. Higher TSK levels were linearly associated with a lower risk of decline in muscle strength and physical performance, whereas the relationship with low muscle mass followed a distinct non‐linear pattern (Figure 6B–D).

**FIGURE 6:**
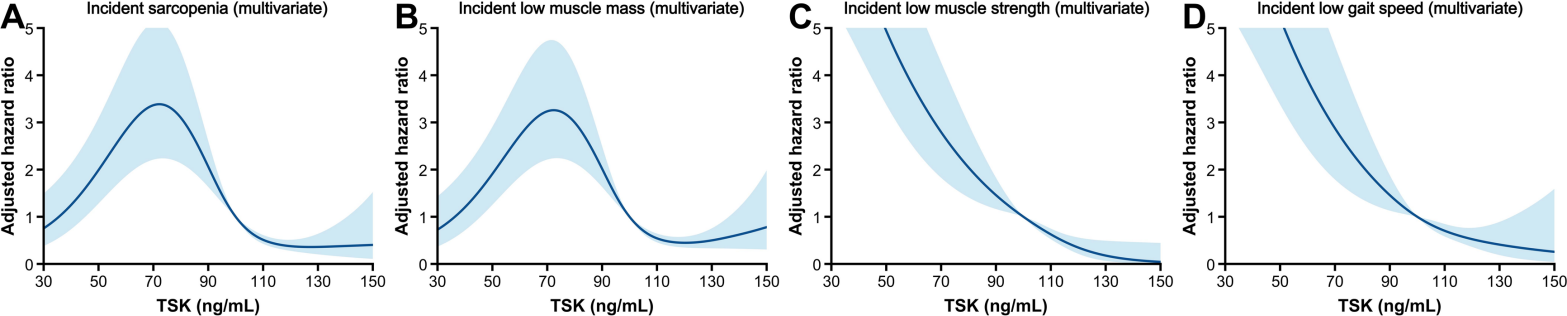
Multivariate‐adjusted restricted cubic spline showing the correlation between serum TSK levels and incidence of sarcopenia (A), low muscle mass (B), low muscle strength (low grip strength) (C) and low physical performance (low gait speed) (D) in Cox proportional hazards models. Model was adjusted for sex, area, BMI, hypertension, ALT, LDL, HDL, ALB and diabetes.

## 4 Discussion

In this prospective cohort study of 1370 community‐dwelling older adults followed for 4 years, we identified circulating TSK as a novel biomarker associated with the development of sarcopenia. Lower baseline serum TSK levels were strongly associated with a higher cumulative incidence of sarcopenia and its components. Moreover, incorporating TSK into a clinical model substantially improved risk prediction, achieving a time‐dependent AUC of 0.808 at 4 years. Taken together, these findings provide the first prospective evidence supporting circulating TSK as a clinically relevant biomarker for early risk stratification of sarcopenia.

TSK is a secreted leucine‐rich repeat protein originally identified as a developmental regulator but more recently recognized as a hepatokine involved in systemic metabolic regulation [4]. Experimental studies have demonstrated that circulating TSK responds to nutritional status and metabolic stress and participates in cross‐organ communication between the liver, adipose tissue and skeletal muscle [3]. Nutritional status and chronic low‐grade inflammation are well‐established determinants of skeletal muscle health in older adults and may influence both circulating hepatokine profiles and the risk of sarcopenia [16]. These overlapping metabolic and muscle‐related processes may partly explain why participants in the highest TSK tertile had better muscle mass and gait speed despite a higher prevalence of diabetes and hypertension. The persistence of the association after excluding participants with baseline diabetes and after adjustment for baseline muscle measures indicates that these measured factors do not fully account for the observed association. Nevertheless, residual confounding cannot be excluded, and circulating TSK may reflect relatively preserved muscle status or broader physiological processes rather than directly protect against sarcopenia. Experimental evidence that TSK contributes to skeletal muscle integrity, exercise endurance and muscle regeneration further supports its potential relevance to muscle physiology [6]. Our findings extend the clinical evidence linking TSK to muscle health by providing longitudinal evidence that circulating TSK is associated with the subsequent development of sarcopenia in older adults. From a clinical perspective, the metabolic properties of TSK may be relevant to its potential utility as a circulating biomarker, given that sarcopenia is influenced by systemic metabolic processes and interactions among multiple organs. The observed association is consistent with the broader concept of liver–muscle communication during muscle aging; however, it does not establish that TSK mediates this communication or that the liver is the primary source responsible for the association. Because hepatic function, nutritional status and inflammatory burden were not comprehensively characterized, their respective contributions cannot be determined from the present data. Future studies integrating detailed hepatic phenotyping, nutritional and inflammatory assessments and mechanistic investigations are needed to clarify whether TSK plays a causal role in sarcopenia development or primarily serves as a marker of systemic metabolic and physiological status.

Another important observation is that the associations between TSK and muscle‐related outcomes may differ by domain. In our component analyses, TSK was positively associated with ASMI and gait speed, whereas no significant cross‐sectional correlation was observed between TSK and baseline handgrip strength. This pattern may be interpreted in light of experimental evidence showing that TSK deficiency in mice is associated with reduced grip strength, impaired endurance capacity and selective disruption of slow‐twitch myofiber gene programmes [6]. Transcriptomic analyses further indicated that TSK deficiency selectively impairs the expression of contractile genes characteristic of slow‐twitch muscle fibres and is associated with abnormal neuromuscular junction coupling [6]. Given the roles of slow‐twitch fibres and neuromuscular connectivity in muscle endurance and physical performance [17, 18], these findings may provide a biological rationale for a potential link between TSK and aspects of muscle function. However, the extent to which TSK influences muscle quality and the underlying mechanisms remain unclear. The absence of a significant correlation between TSK and baseline handgrip strength, despite the inverse association between TSK and subsequent low muscle strength, may reflect differences between concurrent functional status and the risk of future functional decline. Grip strength measured at a single visit may be affected by pain, arthritis, participant effort and other assessment conditions, whereas subsequent functional deterioration may reflect longer term physiological processes. Circulating TSK may therefore capture aspects of systemic physiological status relevant to the maintenance of muscle function rather than directly indicate current muscle strength. This interpretation remains hypothetical, as physiological reserve was not directly assessed in our study. Accordingly, higher circulating TSK levels should not be interpreted as evidence of greater muscle strength at the time of measurement. The AWGS 2025 consensus has further emphasized the importance of muscle health and the preservation of muscle function throughout aging, highlighting the need for earlier identification of individuals at risk of functional decline [19]. Because muscle strength and physical performance may deteriorate before substantial muscle mass loss becomes apparent, biomarkers that capture early metabolic or functional alterations could complement conventional assessments of muscle health. In this context, circulating TSK warrants further investigation as a potential biomarker of future sarcopenia risk. As a metabolically regulated hepatokine potentially involved in interorgan communication, TSK may reflect systemic metabolic processes relevant to skeletal muscle homeostasis [3]. Its association with incident sarcopenia over 4 years supports further evaluation of its potential value in risk stratification among older adults. However, whether TSK improves risk prediction beyond established clinical measures, enables earlier identification of muscle dysfunction or facilitates targeted preventive interventions requires prospective validation in independent cohorts.

A notable finding of our subgroup analyses is that the association between serum TSK and incident sarcopenia appeared to be more evident in women than in men. However, this observation should be interpreted cautiously. The subgroup analyses were exploratory in nature and were not specifically powered to detect sex‐related differences. Therefore, the absence of statistical significance in men does not necessarily indicate a lack of biological association, and the apparent sex‐specific effect may partly reflect limited statistical power. Several biological and metabolic factors may potentially contribute to the observed heterogeneity. Sex differences in hepatic metabolism, body composition, skeletal muscle characteristics and hormonal regulation have all been implicated in muscle aging and may influence hepatokine signalling pathways [20, 21]. In particular, women generally exhibit differences in oxidative muscle fibre composition and experience substantial hormonal changes with aging [22], which could theoretically modify the relationship between circulating TSK and skeletal muscle health. As a hepatokine, TSK production is regulated by hepatic metabolic status and nutritional signals, and sex differences in hepatic lipid metabolism and endocrine signalling may modify its systemic effects on skeletal muscle [3, 23]. Nevertheless, these mechanistic explanations remain speculative and were not directly evaluated in the present study. Accordingly, our findings should be considered hypothesis generating rather than confirmatory. Future studies with larger sample sizes, sex‐specific mechanistic investigations and external validation cohorts are needed to determine whether circulating TSK exhibits true biological heterogeneity across sexes or whether the observed differences are attributable to statistical variability.

## 5 Study Limitations

Several limitations should be acknowledged. First, this was a single‐centre cohort study, which may limit the generalizability of the findings to other populations. Second, serum TSK was measured only at baseline; therefore, longitudinal changes in TSK levels and their relationships with changes in muscle status could not be assessed. Third, residual confounding remains possible despite adjustment for relevant demographic, metabolic, nutritional and clinical covariates. In particular, information on medication use, HbA1c and diabetes duration was unavailable, precluding a more comprehensive assessment of treatment‐related effects and diabetes severity or control. Although no deaths or losses to follow‐up were reported, potential selection bias related to survival before recruitment or the eligibility criteria cannot be excluded. Future studies incorporating detailed treatment histories, more comprehensive assessments of metabolic disease severity and control and repeated measurements of TSK and muscle status are warranted. Fourth, although ALT was included in the multivariable models, it is insufficient to characterize hepatic steatosis or fibrosis. Data required to calculate the fatty liver index (FLI) and fibrosis‐4 index (FIB‐4), as well as liver imaging data, were unavailable. Therefore, we could not adequately characterize underlying liver disease, determine its contribution to the observed association or exclude residual confounding related to hepatic dysfunction. Further studies incorporating comprehensive hepatic phenotyping are needed to clarify the relationship between TSK, liver health and sarcopenia. Fifth, although TSK showed moderate discrimination for incident sarcopenia and the combined clinical model demonstrated improved discrimination, we did not assess additional measures of incremental predictive value, such as the net reclassification improvement (NRI) or integrated discrimination improvement (IDI). Thus, the extent to which TSK improves individual‐level risk classification beyond established clinical predictors remains uncertain and requires further validation. Finally, although our findings support a prospective association between circulating TSK levels and incident sarcopenia, they do not establish causality. Mechanistic studies are needed to clarify the biological pathways underlying this association and to determine whether TSK contributes directly to muscle aging or primarily reflects systemic metabolic and physiological status.

## 6 Conclusions

In summary, in this prospective cohort study of older adults, circulating TSK emerged as a novel hepatokine biomarker that independently predicts incident sarcopenia, particularly functional decline, and significantly improves risk stratification beyond conventional clinical factors. Future multicentre studies and mechanistic studies are needed to validate these findings and to explore whether targeting the liver–muscle axis may represent a novel strategy for the prevention and treatment of sarcopenia.

## Funding

This work was supported by grants from the National Key Research and Development Plan of China (No. 2020YFC2008505 to Xiang Lu); the Project of Zhongda Hospital Affiliated to Southeast University for Cultivating Academic Talent (No. CZXM‐GSPRC22 to Wei Gao); the Zhongda Hospital Affiliated to Southeast University, Jiangsu Province High‐Level Hospital Pairing Assistance Construction Funds (No. ZDLYG10 to Wei Gao); the Zhongda Hospital Affiliated to Southeast University, Jiangsu Province High‐Level Hospital Construction Funds (No. GSP‐LCYJFH17 to Wei Gao); the Research Project on Integrated Traditional Chinese and Western Medicine in Chronic Disease Management (No. CXZH2024140 to Wei Gao); the Major Project of the Jiangsu Provincial Geriatrics Society (No. JGS2025ZDA002 to Wei Gao); the Science and Technology Development Plan Project of Suzhou (No. SKY2023164 to Ming‐Qing He); the Jiangsu Provincial Elderly Health Scientific Research Project (No. LKM2024012 to Ming‐Qing He); the Jiangsu Provincial Cadre Healthcare Scientific Research Project (No. BJ2507 to Ming‐Qing He); the Boxi Clinical Research Project of the First Affiliated Hospital of Soochow University (No. BXLC2024017 to Ming‐Qing He); the Project for Strengthening Health Through Science and Education of Suzhou (No. 2023001312060001 to Ming‐Qing He); the Gusu Health Talent Scientific Research Project of Suzhou (No. GSWS2025013 to Ming‐Qing He); the Chinese National Science Foundation (No. 32271187 to Bin Xue); the Noncommunicable Chronic Diseases—National Science and Technology Major Project (No. 2024ZD0521200 to Bin Xue); the Collaborative Innovation Center for Cancer Personalized Medicine‐Clinical Research Fund of Hengrui Medicine (No. JZ21449020210617 to Bin Xue); the Basic Research Project of Changzhou Medical Center, Nanjing Medical University (No. CMCB202306 to Bin Xue); the Natural Science Foundation of Xinjiang Uygur Autonomous Region (No. 2023D01D05 to Bin Xue); the Top Innovative Talent Program of Nanjing Medical University (No. NJMUTY20230093 to Bin Xue); and the research grant jointly established by the Hope for Rare Foundation and Homy FH China Patient Network (No. FH2025‐023 to Bin Xue).

## Ethics Statement

This study was performed in accordance with the principles outlined in the Declaration of Helsinki and approved by the Ethics Committee of Sir Run Run Hospital, Nanjing Medical University (2019‐SR‐S041). Written informed consent was obtained from each participant.

## Conflicts of Interest

The authors declare no conflicts of interest.

## Supporting information


**Table S1:** Univariable Cox proportional hazards analysis of baseline demographic, clinical and biochemical variables associated with the study outcome.
**Table S2:** Association between baseline serum TSK and incident sarcopenia after excluding participants with baseline diabetes.
**Table S3:** Association between baseline serum TSK and incident sarcopenia with additional adjustment for baseline muscle measurements.
**Table S4:** Association between baseline serum TSK and incident sarcopenia after excluding participants with BMI ≥ 25 kg/m^2^.

## Data Availability

The data that support the findings of this study are available from the corresponding author upon reasonable request.
