# Supplementary material for: Circulating Tsukushi Predicts Incident Sarcopenia and Functional Decline in Older Adults: A Prospective Cohort Study
Source: J Cachexia Sarcopenia Muscle. 2026 Nov 8;17(6):e70423. doi: 10.1002/jcsm.70423 (PMC13745309; doi:10.1002/jcsm.70423)
Supplement: Supplementary file 1 [file JCSM-17-e70423-s001.docx]

Supplementary Tables

**Table S1. Univariable Cox proportional hazards analysis of baseline demographic, clinical, and biochemical variables associated with the study outcome**

| **Variable** | **HR (95% CI)** | ***P* value** |
| --- | --- | --- |
| TSK (per unit) | 0.99 (0.99-1.00) | <0.001 |
| Age | 0.98 (0.96-1.01) | 0.227 |
| Female sex (vs male) | 1.77 (1.24-2.52) | 0.002 |
| Urban area (vs rural) | 0.41 (0.27-0.64) | <0.001 |
| BMI | 0.82 (0.78-0.87) | <0.001 |
| Current smoking | 0.69 (0.41-1.17) | 0.170 |
| Current drinking | 0.81 (0.45-1.46) | 0.479 |
| Hypertension | 0.56 (0.38-0.83) | 0.004 |
| Diabetes | 0.79 (0.48-1.30) | 0.358 |
| FBG | 0.98 (0.89-1.09) | 0.740 |
| ALT | 0.98 (0.96-1.00) | 0.048 |
| AST | 1.01 (0.99-1.03) | 0.384 |
| TBIL | 0.97 (0.94-1.01) | 0.111 |
| eGFR | 1.00 (0.98-1.01) | 0.688 |
| BUN | 1.08(0.96-1.21) | 0.188 |
| TC | 1.01 (0.83-1.23) | 0.891 |
| TG | 0.76 (0.55-1.06) | 0.104 |
| LDL | 0.72 (0.55-0.93) | 0.012 |
| HDL | 2.26 (1.27-4.04) | 0.006 |
| ALB | 0.96 (0.94–0.99) | 0.003 |
| hs-CRP | 1.07 (0.97-1.17) | 0.202 |

HRs and 95% CIs were estimated using Cox proportional hazards regression based on baseline variables.

**Table S2. Association between baseline serum TSK and incident sarcopenia after excluding participants with baseline diabetes**

| **Population** | **n** | **Events (n)** | **Unadjusted HR (95% CI)** | ***P* value** | **Adjusted HR (95% CI)** | ***P* value** |
| --- | --- | --- | --- | --- | --- | --- |
| Full cohort | 1,370 | 126 | 0.74 (0.63–0.87) | <0.001 | 0.64 (0.53–0.76) | <0.001 |
| Without baseline diabetes | 1,133 | 108 | 0.74 (0.62–0.89) | 0.001 | 0.63 (0.52–0.77) | <0.001 |

HRs are expressed per 1-SD increase in baseline serum TSK. The full-cohort sample SD (31.73 ng/mL) was used in all models. Cox proportional hazards models used the Efron method for tied event times; 95% CIs and two-sided *P* values were based on Wald statistics.

The full-cohort adjusted model included sex, residential area, BMI, hypertension, ALT, LDL, HDL, ALB, and diabetes. The analysis restricted to participants without baseline diabetes used the same covariates except diabetes. Both unadjusted models included TSK only.

ALB, albumin; ALT, alanine aminotransferase; BMI, body mass index; CI, confidence interval; HDL, high-density lipoprotein cholesterol; HR, hazard ratio; LDL, low-density lipoprotein cholesterol; SD, standard deviation; TSK, Tsukushi.

**Table S3. Association between baseline serum TSK and incident sarcopenia with additional adjustment for baseline muscle measurements**

| **Model** | **Adjusted HR (95% CI)** | ***P* value** |
| --- | --- | --- |
| Primary model | 0.64 (0.53–0.76) | <0.001 |
| Primary model + baseline ASMI | 0.64 (0.53–0.77) | <0.001 |
| Primary model + baseline handgrip strength | 0.64 (0.53–0.76) | <0.001 |
| Primary model + baseline gait speed | 0.64 (0.54–0.77) | <0.001 |
| Primary model + baseline ASMI, handgrip strength, and gait speed | 0.64 (0.53–0.77) | <0.001 |

HRs are expressed per 1-SD increase in baseline serum TSK. The full-cohort sample SD (31.73 ng/mL) was used in all models. Cox proportional hazards models used the Efron method for tied event times; 95% CIs and two-sided P values were based on Wald statistics.

The primary model was adjusted for sex, residential area, BMI, hypertension, ALT, LDL, HDL, ALB, and diabetes. Baseline ASMI (kg/m²), handgrip strength (kg), and gait speed (m/s) were entered as continuous linear covariates, individually or simultaneously, as indicated. The three individual-adjustment models each add one measure to the primary model and are not cumulative.

ALB, albumin; ALT, alanine aminotransferase; ASMI, appendicular skeletal muscle mass index; BMI, body mass index; CI, confidence interval; HDL, high-density lipoprotein cholesterol; HR, hazard ratio; LDL, low-density lipoprotein cholesterol; SD, standard deviation; TSK, Tsukushi.

**Table S4. Association between baseline serum TSK and incident sarcopenia after excluding participants with BMI ≥25 kg/m²**

| **Population** | **n** | **Events (n)** | **Unadjusted HR (95% CI)** | ***P* value** | **Adjusted HR (95% CI)** | ***P* value** |
| --- | --- | --- | --- | --- | --- | --- |
| Full cohort | 1,370 | 126 | 0.74 (0.63–0.87) | <0.001 | 0.64 (0.53–0.76) | <0.001 |
| BMI <25 kg/m² | 1,005 | 116 | 0.68 (0.57–0.80) | <0.001 | 0.57 (0.47–0.69) | <0.001 |

HRs are expressed per 1-SD increase in baseline serum TSK. The full-cohort sample SD (31.73 ng/mL) was used in all models. Cox proportional hazards models used the Efron method for tied event times; 95% CIs and two-sided P values were based on Wald statistics.

Adjusted models included sex, residential area, BMI, hypertension, ALT, LDL, HDL, ALB, and diabetes. BMI was retained as a continuous covariate in the restricted analysis because it continued to vary among participants with BMI <25 kg/m². Both unadjusted models included TSK only.

ALB, albumin; ALT, alanine aminotransferase; BMI, body mass index; CI, confidence interval; HDL, high-density lipoprotein cholesterol; HR, hazard ratio; LDL, low-density lipoprotein cholesterol; SD, standard deviation; TSK, Tsukushi.
